# The volatile compound BinBase mass spectral database

**DOI:** 10.1186/1471-2105-12-321

**Published:** 2011-08-04

**Authors:** Kirsten Skogerson, Gert Wohlgemuth, Dinesh K Barupal, Oliver Fiehn

**Affiliations:** 1 Genome Center, University of California, Davis, CA 95616 USA

## Abstract

**Background:**

Volatile compounds comprise diverse chemical groups with wide-ranging sources and functions. These compounds originate from major pathways of secondary metabolism in many organisms and play essential roles in chemical ecology in both plant and animal kingdoms. In past decades, sampling methods and instrumentation for the analysis of complex volatile mixtures have improved; however, design and implementation of database tools to process and store the complex datasets have lagged behind.

**Description:**

The volatile compound BinBase (vocBinBase) is an automated peak annotation and database system developed for the analysis of GC-TOF-MS data derived from complex volatile mixtures. The vocBinBase DB is an extension of the previously reported metabolite BinBase software developed to track and identify derivatized metabolites. The BinBase algorithm uses deconvoluted spectra and peak metadata (retention index, unique ion, spectral similarity, peak signal-to-noise ratio, and peak purity) from the Leco ChromaTOF software, and annotates peaks using a multi-tiered filtering system with stringent thresholds. The vocBinBase algorithm assigns the identity of compounds existing in the database. Volatile compound assignments are supported by the Adams mass spectral-retention index library, which contains over 2,000 plant-derived volatile compounds. Novel molecules that are not found within vocBinBase are automatically added using strict mass spectral and experimental criteria. Users obtain fully annotated data sheets with quantitative information for all volatile compounds for studies that may consist of thousands of chromatograms. The vocBinBase database may also be queried across different studies, comprising currently 1,537 unique mass spectra generated from 1.7 million deconvoluted mass spectra of 3,435 samples (18 species). Mass spectra with retention indices and volatile profiles are available as free download under the CC-BY agreement (http://vocbinbase.fiehnlab.ucdavis.edu).

**Conclusions:**

The BinBase database algorithms have been successfully modified to allow for tracking and identification of volatile compounds in complex mixtures. The database is capable of annotating large datasets (hundreds to thousands of samples) and is well-suited for between-study comparisons such as chemotaxonomy investigations. This novel volatile compound database tool is applicable to research fields spanning chemical ecology to human health. The BinBase source code is freely available at http://binbase.sourceforge.net/ under the LGPL 2.0 license agreement.

## Background

Volatile compounds comprise diverse chemical groups with wide-ranging sources and functions. They are typically small compounds (ranging from C5 to C20 carbon count) with a molecular weight maximum of approximately 500 Daltons (C35), and have low boiling points and high vapor pressures at ambient temperature and pressure [1,2]. Volatile compounds originate from major pathways of secondary metabolism of many organisms and play important, varied roles in chemical ecology in both plant and animal kingdoms [3]. From a commercial standpoint, volatile compounds are critical in flavor and fragrance industries and for food authentication and quality measures [4,5]. Additionally, volatile measures from skin and breath are being increasingly used in disease detection and diagnosis [6,7]. In the plant kingdom alone, several thousand volatiles have been identified from floral, vegetative and fruit tissues [8]. These compounds comprise 1% of plant metabolites and include terpenoids, phenylpropanoids, benzenoids, amino acid derivatives, and fatty acid derivatives [9]. Because of the role volatiles play in plant pollination and defense as well as fruit quality, there is much interest in identifying individual components in the complex mixtures and elucidating biosynthetic pathways to target in breeding programs [10]. Additionally, because biotic and abiotic factors affect the identity, quantity and timing of their release, plant volatiles could potentially serve as biomarkers of a plant's physiological and disease states. Recent work has demonstrated applications for volatile biomarkers including monitoring fruit maturity [11], detecting the presence of herbivore or microbial pests [12,13], and monitoring disease or water stresses [14]. Not only identity, but relative quantities may be important in defining volatile signatures. Researchers' ability to extract and decipher important signals or biomarkers from complex mixtures will depend on the ability to track and catalog hundreds of compounds over thousands of samples, sample types and studies [3,15]. Researchers in fields spanning chemical ecology, flavor and fragrance chemistry, and medicine require better database and library tools capable of tracking and identifying compounds in complex volatile mixtures.

In past decades, technical developments in volatile compound sampling devices including mixed-phase solid phase microextraction (SPME) fibers, multi-phase sorbent tubes, radial samplers, and the Twister™ stir bar sorptive extraction (SBSE) device have enhanced the ability to capture volatile compounds under static and dynamic sampling conditions [16-20]. Separation and detection of these complex mixtures is routinely performed by gas chromatography-coupled mass spectrometry (GC-MS). Time-of-flight mass spectrometers (TOF-MS) are particularly well-suited for these analyses, as the high-speed spectral acquisition and absence of spectral skew allow for reliable peak deconvolution of co-eluting compounds. Two-dimensional GC (GCxGC-TOF-MS) is increasingly employed to improve signal-to-noise ratios, peak resolution, and subsequent compound identification [21-23]. Despite these advances in sampling and detection, few reports describe innovations in annotation and database tools or data analysis strategies to handle these large, complex datasets. The development of better data processing methods remains an unmet need in volatile compound research.

In response to the needs of the metabolomics community, a number of software programs have been developed to address the issues surrounding automated, consistent analyses of complex GC-MS datasets. Many tools, including AMDIS [24], SpectConnect [25], MZmine [26], TagFinder [27] and MetAlign [28] are freely available. These programs support peak identification, chromatographic alignment, library-based assignments, batch processing, and report generation in formats suitable for further processing with statistical software. All are programmed to annotate unknown compounds (i.e., compounds not present in the interfaced library) within a particular experiment, but none incorporates a database approach that allows for the tracking of these unknown compounds in subsequent studies based on their mass spectrum and retention index.

Separate from the software tools mentioned above, volatile compound mass spectral libraries and databases are available to aid in compound identification. Annotation of mass spectra generated by electron ionization requires two independent parameters, as the electron ionization (EI) spectra of stereoisomers and positional isomers are often nearly identical. The Kovats retention index (RI)--a standardized chromatographic retention time based on alkanes--is most commonly used as the second parameter for compound annotation [29]. Despite this, only select commercial libraries such as the Robert Adams [30] and Mass Finder Terpenoids [31] libraries report Kovats RI values for all compounds. Publically accessible volatile compound databases [32-34] contain Kovats RI information, but these manually curated collections suffer several limitations. Most were built to target a specific research area and are limited in scope. Furthermore, these compound databases are not available in formats that can be integrated into annotation tools for automated mass spectral matching.

The database we present here is therefore different from commonly used tools in the field of Analytical Chemistry: it is not only a library or a data processing tool, but combines these capabilities in a much advanced, comprehensive repository. There are many differences between a 'library' and a 'database'. Generally, databases provide extended capabilities of using a lot more input information (called 'metadata') and giving a large array of output information, and (linked) query options. For our previously established BinBase database of derivatized (primary) metabolites [35,36], we have shown how to use the plethora of mass spectral metadata as input obtained from deconvoluted spectra (i.e. retention indices, unique ions, apex ions, peak signal-to-noise ratios, and peak purities) to annotate peaks from sets of chromatograms to database entries ('bins') that are defined by these metadata. Such bins are assigned by chemical entities using libraries (e.g. the Fiehnlib libraries [29] or the NIST08 library). By default, users then obtain coherent data sheets of annotated compounds that comprise a lot more output information than using classic vendor-based software, such as: automatic links to chemical and biochemical databases, full mass spectra for all detected compounds, observed retention indices for each peak, different output data sheets constraining the number of reported compounds by the percentage of positive peak detections per experimental class, and web-based query options that allow users to query compounds across all studies and matching spectra against all spectra within BinBase.

However, our classic BinBase repository was tailored to trimethylsilylated (primary) metabolites and could not be directly used to process chromatograms from underivatized volatiles. We here present here the expansion of the existing metabolite BinBase database to track and identify volatile compounds in complex and diverse mixtures, in tens or thousands of samples, regardless of sample source. This repository represents the first publicly available, large cohort of volatile compound profiles with associated mass spectra files and source code as freely available downloads.

## Construction and Content

### Volatile sample collection and analysis

#### Volatile compound sampling

**Figure 1:**
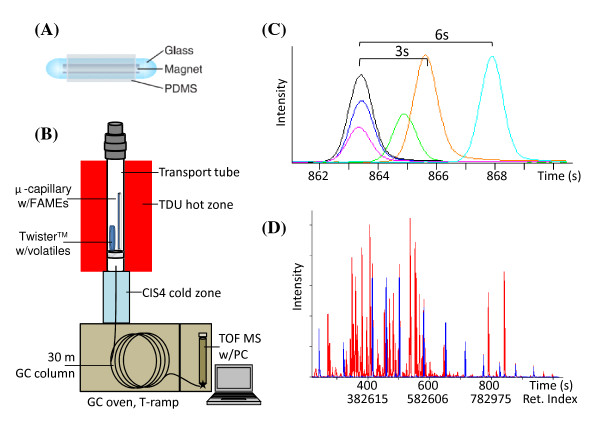
**Volatile compounds are captured using Twister™ technology and detected by GC-TOF-MS with a time-invariant FAME-based retention-index grid**. *(A) Sample collection*. Volatile compounds are trapped on 1 cm long PDMS-coated Twisters™. (Figure provided by GERSTEL, Inc.). *(B) Schema of data acquisition instrumentation *(not to scale). Exposed Twisters™ are transferred into glass transport tubes together with an external solution of retention index markers (C4-C26 fatty acid methyl esters, FAMEs) in 0.5 ml glass capillaries. Volatiles are released from the Twisters™ in a thermal desorption unit (TDU). Desorbed volatiles and FAMEs are refocused in the liquid nitrogen-cooled CIS4 inlet, then re-volatilized for temperature-ramped separation on a gas chromatography (GC) column for electron ionization time of flight mass spectrometry detection (TOF MS) and primary data processing on ChromaTOF software. *(C) Retention time shifts*. Over long periods of operation, absolute retention times (RT) of compounds drift due to column use. Shown here is the RT shift for methyl eicosanoate (C20 FAME) from six separate samples during a two-year study covering 1,500 samples. Shifts of 3 seconds occurred during one month of operation, while a 6 second shift was observed after a column change. *(D) Conversion to retention index*. Adding FAME retention index markers (m/z 74, 5-fold magnified, blue trace) to every volatile profile (total ion chromatogram, red trace) establishes a stable grid of FAMEs to convert variable 'time' into invariable 'index' units. No further chromatogram alignments are needed.

Volatile compound sampling protocols (sorbent choice and sampling method) are specific to analyte identity and sample source, and vary widely depending on the research area and focus. The majority of our sampling has employed the polydimethylsiloxane (PDMS)-based TwisterTM (GERSTEL, Inc.) because of its high capacity, versatility (both headspace and stir-bar sorptive extraction modes possible) and ease-of-handling in field settings (Figure 1A). Volatile compounds captured by the TwisterTM are thermally desorbed for analysis (Figure 1B). Although TwistersTM have been our primary sorbent to date, other sorbent types and volatile sampling methods (e.g., packed cartridge, SPME, direct headspace injections and direct thermal desorption) can be used and are compatible with data annotation and Bin databasing.

#### Retention index markers

Absolute retention times (RT) of GC-MS peaks shift as a function of column properties (e.g., column type, age, length, phase ratio, film thickness) and RT differences are frequently observed among samples or sample types (Figure 1C). When performing large studies spanning months or years, or comparing many different sample types, RT shifts are unavoidable. Retention indices (RI) overcome this problem by locking the retention times of eluted compounds to fixed positions defined by marker compounds spiked into the sample. Highly different samples can be compiled in a database over years with the use of RI markers.

The vocBinBase algorithm requires the addition of RI marker compounds to all samples for RI corrections. We use fatty acid methyl esters (FAMEs) as RI markers rather than classic straight-chain alkanes (Kovats RI) because FAMEs exhibit electron ionization (EI) fragment patterns (especially at high m/z values) better suited for unambiguous and automated detection. To avoid confusion between the FAME-based RI values and Kovats-based RI values (carbon number * 100), we have adopted a distinctive unit value and FAME RI values range from 262,214 for FAME C4 to 980,934 for FAME C24. For reference, the corresponding alkane-based RI values for FAMEs C4 and C24 are 726 and 2712, respectively. Both FAMEs and alkanes are naturally occurring volatiles [8], so the addition of the RI mixture will prevent the detection of the specific marker compounds added unless isotopically labeled RI markers are used.

The RI mixture for volatile samples includes FAMEs of linear carbon chain lengths C4, C6, C8, C9, C10, C12, C14, C16, C18, C20, C22, and C24. A stock mixture is prepared in methylene chloride with final FAME concentrations of 5 mg/mL (C4), 1.5 mg/mL (C20, C22, C24), 1.2 mg/mL (C6, C8), 0.8 mg/mL (C9, C16, C18) and 0.4 mg/mL (C14-C18). This FAME stock solution is then diluted 200-fold in methyl propionate prior to use. The working FAME RI mixture is introduced externally to the Twister™ in 0.5 uL capillaries. Capillaries are filled with the FAME RI solution and then placed alongside the Twister™ in a frit-bottomed TDU transport tube for thermal desorption (Figure 1B). Chromatograms illustrating the grid-like nature of the FAME RI markers in a citrus leaf volatile sample spiked using the capillary method are shown below (Figure 1D).

#### Instrumentation

Volatile sample analyses are performed on a 6890 GC (Agilent Technologies, Santa Clara, CA) equipped with a thermal desorption unit (TDU, GERSTEL, Inc., Muehlheim, Germany), cryo-cooled injection system inlet (CIS4, GERSTEL, Inc.) and robotic sampler (MPS2, GERSTEL, Inc.) interfaced to the Pegasus IV time-of-flight mass spectrometer (Leco, St. Joseph, MI).

#### Thermal desorption and injector parameters

Exposed Twisters are thermally desorbed in the TDU in splitless mode (50 mL/min flow rate, solvent vent mode) at an initial temperature of 30°C, ramped to 250°C at a rate of 12°C/sec, and then held at the final temperature for 3 min. The desorbed analytes are cryofocused in the CIS4 inlet with liquid nitrogen (-120°C). After desorption the inlet is heated from -120 to 260°C at a rate of 12°C/s and held at 260°C for 3 min.

#### GC-TOF-MS settings

GC-TOF-MS instrument settings and programming are defined in standard operating procedures in order to produce data that can be auto-annotated and compiled across studies. Chromatographic separation is performed on an Rtx-5SilMS column with a 10 m integrated guard column [95% dimethyl/5% diphenyl polysiloxane film; 30 m × 0.25 mm (inside diameter) × 0.25 μm d.f. (Restek, Bellefonte, PA)]. The GC oven temperature program is as follows: initial temperature of 45°C with a 2 min hold followed by a 20 °C/min ramp up to 300°C with a 2 min hold followed by a 20 °C/min ramp up to 330°C with a 0.5 min hold. The carrier gas (99.9999% He) flow is held constant at 1 mL/min. The transfer line temperature between the gas chromatograph and mass spectrometer is 280°C. Mass spectra are acquired at 25 spectra/sec with a mass range of 35-500 m/z. The detector voltage is set at 1800 V and the ionization energy at 70 eV. The ion source temperature is 250°C.

### Binbase database construction

#### Database structure

**Figure 2:**
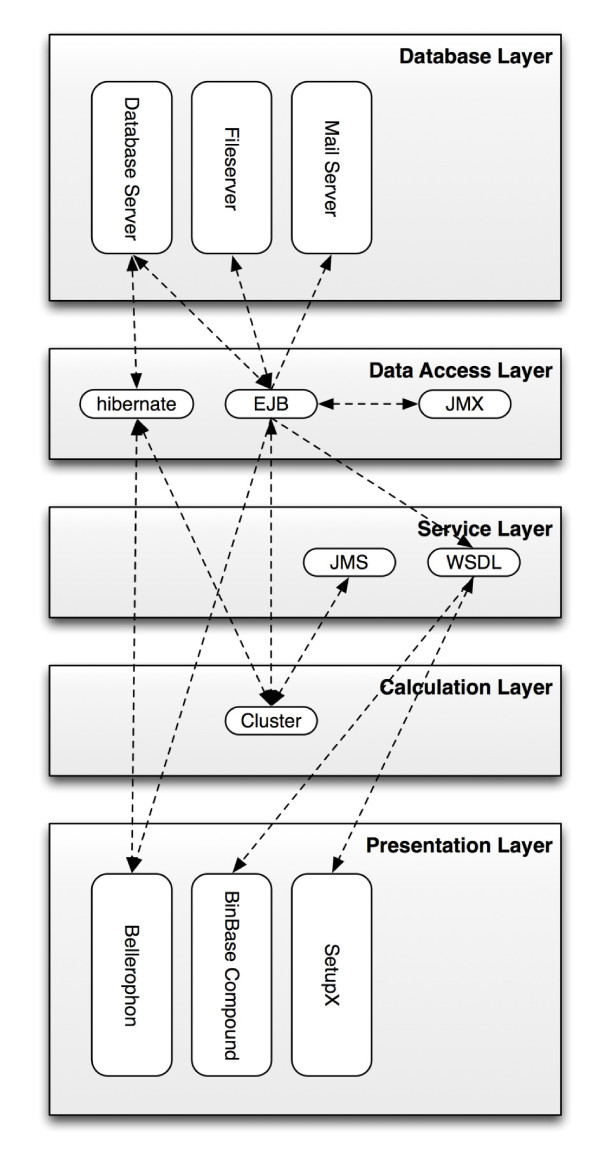
**Multilayered architecture of the vocBinBase Database**.

The BinBase code was developed in Java and Groovy, and is based entirely on open-source software. BinBase employs multilayered software architecture (Figure 2). At the core of BinBase is an SQL-conforming database which stores mass spectra (generated during sample analysis), analysis results and cached data (for improved speed). Database contents are accessed by the cluster, application server and Bellerophon using Java Database Connectivity (JDBC). This access is encapsulated by Enterprise JavaBeans (EJB) and the Hibernate Object mapping framework. The BinBase central configuration is stored in the Application Server, which also houses EJB, WSDL (Web Service Description Language)-based services, JMS (Java Messaging Service), and JMX (Java Management Extensions) components; together these comprise the BinBase Communication Interface (BCI). These EJBs provide an interface to the database and allow other Java programs to access the database, query data and start calculations in a defined, restricted manner. The Hibernate persistence and object mapping layer allows for execution of complex queries in a simple, intuitive way and is primarily used by Bellerophon, the BinBase administration graphical user interface (GUI) (see below). A WSDL service layer was added to overcome EJB limitations so that BinBase can be accessed from most programming languages. Internally, the WSDL service layer is also used for all web front-ends and communications with SetupX/MiniX. JMX components are used to configure the whole system at a central location and monitor system properties. The BCI module plays a key role in system security by limiting user access to particular services based on IP address and password, and by preventing denial of service (DoS) attacks or SQL injection attacks.

#### BinBase database installation requirements

The BinBase system requires a Rocks Linux cluster-based architecture to calculate mass spectral data. This is minimally established with a system consisting of two standard personal computers (PC's). The first PC stores data (*.netcdf files,*.txt files and database content), provides access to web pages and maintains the calculation queue. The second PC performs calculations. A dual core 2 GHz central processing unit (CPU) and 4 GB RAM are sufficient for each of these PC's if the calculation load does not exceed several hundred samples a day. Because of its data storage function, the first PC requires 1-2 TB storage and two 1 GB network cards. A smaller hard drive (200 GB) and a single network card are sufficient for the second PC. Our current configuration at the Genome Cente' each and one head node with a solid state disk-based storage array for improved database access.

The BinBase database is available to the public under the LGPL 2.0 license (http://binbase.sourceforge.net), and is accessible using different web front-ends and rich client applications as well as a webservice layer. Documentation required for installation and administration of the system is also found at this website.

#### Bellerophon

The front-end graphical user interface (GUI) Bellerophon is the central administration tool for BinBase and is used for Bin management, database browsing and retention index configuration. Bellerophon is an Eclipse 3 SWT-based rich client platform (RCP) application. It includes visualization capabilities based on JFreeChart and supports database queries via a Hibernate framework. The Hibernate framework supports mapping database tables to objects. Dynamic SWT-tables and visualizations are created from these objects via Java Reflection-API and XDoclet.

#### SetupX

SetupX is a study design database whose primary functions include capturing experimental metadata for class generation, randomizing and scheduling GC-TOF-MS sequences, and storing annotated GC-TOF-MS data along with all other data files connected to an experiment (e.g., photographs, assay spreadsheets, other instrumental data files). Details regarding SetupX structure have been described [35,37]. We have developed a leaner version of this database, MiniX. User requests for BinBase annotations through the MiniX website activate the MiniX BinBase export function by EJB and JMS. BinBase additionally requests experimental class information from MiniX through EJBs. MiniX is an open source project and can be downloaded and installed under the LGPL 2.0 license (http://code.google.com/p/minix/).

#### vocBinBase filtering algorithm

The vocBinBase algorithm takes the deconvoluted spectra and metadata provided by the Leco ChromaTOF software as well as sample information from the study design database SetupX/MiniX and applies a multi-tiered filtering system that either annotates spectra to existing database entries ('Bins'), creates and adds new Bins to the database if all quality criteria are met, or discards low-quality spectra to maintain database integrity (see Additional File 1, figure S1). Each database entry or "Bin" represents a unique compound that has matched all mass spectral, instrumental and class metadata thresholds. Bins are minimally defined by the following properties: mass spectrum, retention index (RI), quantification mass, list of unique masses, and a unique identifier number.

#### Data preprocessing

Raw data are pre-processed by the Leco ChromaTOF software and stored as ChromaTOF-specific *.peg files, generic *.txt results, and as generic ANDI MS *.cdf files. ChromaTOF (v. 2.32) data processing parameters specified in pre-processing steps include baseline setting just above noise (value = 1), no smoothing, and signal-to-noise ratio minimum of 20. The *.txt files are exported to a file server for further processing by the algorithm. The vocBinBase algorithm is compatible with ChromaTOF software versions 2.32 to the current version, 4.33.

#### Spectral validation

After importing all deconvoluted spectra of all chromatograms of a biological study (*.csv format), spectra are checked for the presence and abundance of the unique ion (relative to the base peak), the presence of all apex masses (masses that share the maximum intensity with the peak maximum of the unique ion), and for the number of peaks that exceed apex intensity thresholds. Spectral validation is the first data quality filter; chromatograms with overloaded peaks and deconvolution errors are used only for peak matching, but not for Bin generation.

#### Retention index calculations based on fatty acid methyl esters

The BinBase algorithm for retention index correction first applies a base peak filter to all spectra to locate the FAME RI markers (no retention time information is used). From this filtered list, the FAME peak with the highest mass spectral similarity score is used as the reference point from which distance measures are applied to higher and lower retention times to locate all other RI markers. Once all the required FAME markers are found, a correction curve is calculated using a linear regression for the first two and last two standards and a polynomial regression of the fifth-order for the standards in between. The polynomial regression is applied within the calibrated range to account for the absolute and relative retention time shifts, which differ from linear regressions at early and at late retention times. As high-degree polynomials perform poorly at extrapolating, linear regression is used to extrapolate outside the RI marker range. In the event that not all early- and late-eluting RI markers are found, the generation of new Bins is disabled, but matching existing Bins is still viable.

Parameters used to find the RI markers for volatile samples required substantial modification from those used in the metabolite algorithms. Match settings and base peak patterns had to be redefined to accommodate the extension of the FAMEs to include C4 and C6, as well as the change in the m/z range from 85-500 to 35-500. This extension of the m/z range to lower values is absolutely required for the volatile compounds, as they are not TMS-derivatized and the 35-85 m/z range provides important fragment data to aid in compound identification. To avoid losing high quality data in which FAMEs were not in specification, existing algorithms were modified to allow for the application of a correction curve of a previous or later sample acquired on the same day to the sample in question. If no such valid RI data were found, search windows were extended up to ten days; otherwise, a partial curve is generated using the RI markers found in the solitary sample. In all of these cases, Bin generation is disabled, but all existing Bins are assigned.

#### Peak annotation by the BinBase algorithm

**Figure 3:**
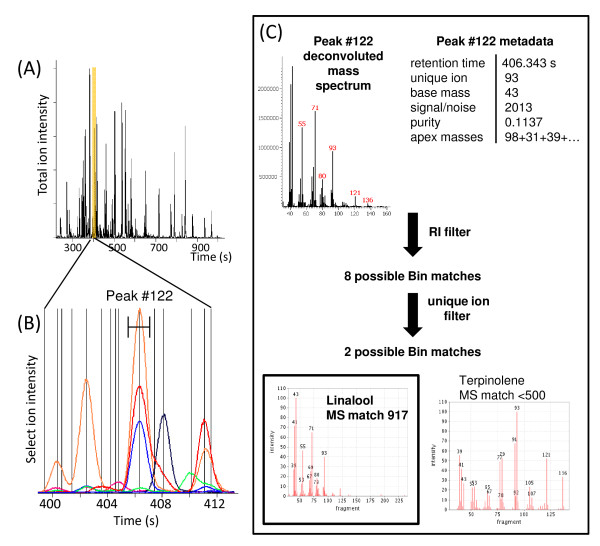
**Example demonstrating the filtering algorithm**. Volatile data collected from the headspace of a wounded orange leaf is complex (A) and spectral deconvolution is required to resolve overlapping peaks. An overlay of 7 out of 465 measured mass elution profiles (m/z 93, 111, 114, 115, 132, 136, 150) is shown from 400-412 seconds (B). Deconvoluted mass spectra and peak metadata are fed into the algorithm. The first two filters use RI information and unique ion information. These are very effective at narrowing database match possibilities as shown for Peak #122 (C).

The ChromaTOF metadata used in peak annotation by the BinBase algorithm include mass spectral similarity, peak purity (an estimate of the number, proximity and similarity of co-eluting peaks), retention index, signal-to-noise ratio, unique ion, apex ions and unique mass-to-base peak ratio. Additional metadata reported by the ChromaTOF software (e.g. peak height, area %) are not used by the algorithm. Following RI correction (described above), spectra are sequentially annotated by decreasing peak intensity. For a given peak, the algorithm sets an RI window (± 2,000 FAME RI units, ~2 sec) and uses a unique ion match filter to match either the unique ion or apexing ions of the deconvoluted peak to generate a list of possible Bin assignments. With just these two parameters, a high degree of filtering is achieved. For example, a compound with a FAME RI value of 446700 and the unique ion m/z 93, the RI filter constraints reduce the number of mass spectra comparisons from 1,537 entries to eight potential hits. The unique ion constraint further reduces possible Bin matches from eight hits to two candidates [terpinolene (monocyclic terpene) or linalool (linear terpene alcohol)] (Figure 3). Only at this stage is a mass spectral similarity filter applied, which uses variable thresholds based on peak signal-to-noise ratio and peak purity. An abundant, well-resolved peak requires a higher mass spectral similarity score for successful annotation than a small or co-eluting peak.

In effect, different thresholds for each parameter can be defined for different peaks. In the example illustrated above (Figure 3), the peak is reasonably pure (peak purity = 0.1137) and a high mass spectral similarity score is required for Bin matching. Based on these final filtering criteria and the mass spectral similarity scores for linalool (917) and terpinolene (<500), the final compound assignment in this example is linalool. In this particular example, there are, in fact, three Bins within the ± 2000 FAME RI unit window, two which have a unique ion value of m/z 93. This second Bin with the unique ion m/z 93 is, in fact, terpinolene.

At this stage in the annotation, more than one Bin assignment may remain (*e.g*., stereoisomers that might elute within the search RI window). The isomer with the closest matching RI is then annotated, unless an alternate Bin has a significantly greater similarity score. Spectra that are filtered out in the isomer filter might still be able to match other neighboring Bins and are therefore fed back into the annotation algorithm.

#### New Bin generation - tracking unknown compounds

In the event the spectrum does not match an existing Bin, the BinBase algorithm generates a new Bin if specific, highly stringent criteria are met. First, the spectrum in question must pass strict mass spectral quality thresholds based on purity (purity value < 1.0) and intensity (S/N > 25). Thresholds for the Bin-generating mass spectral filter are more stringent than those for the similarity filter to ensure that only abundant and pure spectra become new Bins. Second, a potential new Bin must pass an experimental class filter before being validated. This filter demands that a new Bin is detected in at least 80% of all samples of an experimental class in order to ensure its identity as a genuine volatile and not a spurious contaminant. All database Bins were generated by the algorithm as described from data collected in laboratory and field experiments.

#### Post-matching and replacements

Once all spectra of all experimental classes have been annotated, a comprehensive Bin list including all Bins found across the experiment is compiled. Then all spectra are again matched against the Bin list (post-matching) in order that all Bins, including any newly-generated Bins, are searched in all samples. In this step, spectra in samples which did not pass the more stringent MS thresholds required for Bin generation may pass the thresholds required for Bin annotation.

In some cases a Bin is not positively detected in all chromatograms either because it is absent or is low abundant (true negative), or it is present but the quality criteria are not sufficient to allow assignment (false negative). This would result in a zero value in the data matrix, which hampers subsequent statistical analyses. A strategy has been devised and programmed into the algorithm to calculate a replacement value in these cases. First the algorithm determines the average retention time for each metabolite over the analytical sequence by calculating the average retention index for the samples and transforming it back to the retention time using the retention index correction curve. Next the raw, unprocessed chromatograms (netCDF or ANDI MS file formats) are opened and the maximum ion intensity at the select quantification ion trace for each missing volatile compound at ±2s around the target retention time is reported minus the local background noise for that target ion at ±5s around the target retention time. The background subtracted ion intensity is reported in the result table with color coding to indicate the results as a 'second-pass' assignment. Validation of the replacement algorithm was performed by comparing manual annotations of replaced values in sample sets with their algorithm replacement values.

#### vocBinBase Report

All Bins detected in at least 80% of an experimental class are included in the result report folder. Additionally, the report folder contains a result file for all Bins detected in at least 50% of an experimental class. The 50% result can be used by researchers to complement the 80% dataset with more identified metabolites or to evaluate the less confidently found or rare peaks. Each entry in the exported Bin table is reported as the intensity of the Bin quantifier mass, which is by default the unique ion, though this value can be changed manually to any ion in the spectrum by the database administrator. We use peak heights and not peak areas for several reasons. Peak heights are preferable to peak areas for small peaks, because baseline settings impact peak areas more for small peaks than for larger peaks. Additionally, peak heights based on defined unique ions provide a more stable measure than other parameters such as dTIC or TIC, because for analyzing a given compound in different chromatograms, the number and hence, the combined intensity of detected ions will differ, depending on the peak abundance and purity.

**Figure 4:**
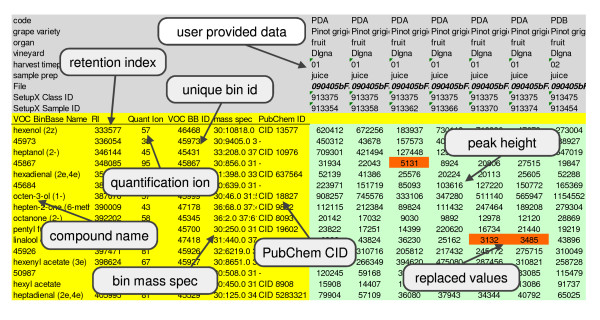
**Sample vocBinBase report highlighting report features**. All Bins exported by the vocBinBase database are reported with a unique database identifier, the quantification ion, retention index and the complete mass spectrum encoded as string. Compound abundances are reported as the intensity of the Bin quantifier mass. Database entries are named using the Adams plant volatile library and hyperlinked PubChem identifiers are included for identified compounds.

All Bins exported by the vocBinBase database are reported with a unique database identifier, the quantification ion, the retention index value, and the complete mass spectrum encoded as a string (Figure 4). Database entries are named using the Adams plant volatile library (described below). Compounds that are not plant-derived including pesticides, plasticizers and other contaminants are annotated using the NIST-RI library. Known artifacts related to column bleed are annotated in vocBinBase, but are not exported to users in result reports (m/z 207, 221, 281, 355). Database administrators can manually exclude (or include) peaks in the list of reported Bins. For example, Twister™-based artifacts are manually selected for exclusion in results tables. Result data sheets are produced as XLS and TXT formats (or XML if needed). Once identified, Bins are also reported with their chemical name and PubChem identifier.

#### Bin Identification

Bin identification is supported by the Adams library of mass spectra and retention index data for over 2,000 purified plant volatiles and essential oil components [30], verified for many compounds using authentic standards in our laboratory. Prior to uploading the Adams library into Bellerophon for Bin matching the library was converted from HP Chemstation format to NIST library format by the Lib2NIST download available at the NIST website (http://chemdata.nist.gov). Additionally, the alkane-based Adams RI values were converted to their BinBase FAME RI equivalent. The RI conversion between the Adams and Fiehn chromatographic variants (different GC oven temperature programming and column manufacturer) was accomplished with a 2^nd^-order polynomial and are given at http://fiehnlab.ucdavis.edu/projects/VocBinBase/. All identified volatiles in vocBinBase are annotated with PubChem chemical identifiers and structure-encoding InChI hash keys to enable cross-references to chemistry databases and structural information tools.

The quality of the RI conversion was tested by injecting authentic reference standards present in the Adams library under standard operating parameters. A comparison of the calculated values with experimentally determined values for 70 reference compounds yielded a correlation of 0.9995 with a standard error of 3,380 RI units (standard deviation of residual error, RI_calculated_-RI_experimental_). A comparison of calculated and experimental values for 130 Adams library annotations yielded similar values (r^2 ^= 0.9994, SE = 3,320 RI units). A plot of the absolute RI deviation (RI_calculated_-RI_experimental_) for the 70 standards and 130 library annotations revealed that 61% of the injected compounds were within one standard error, and 58% of the annotated compounds fell within one standard error of the calculated value. See Additional File 2, figure S2 for the graphed data.

#### Database contents

**Figure 5:**
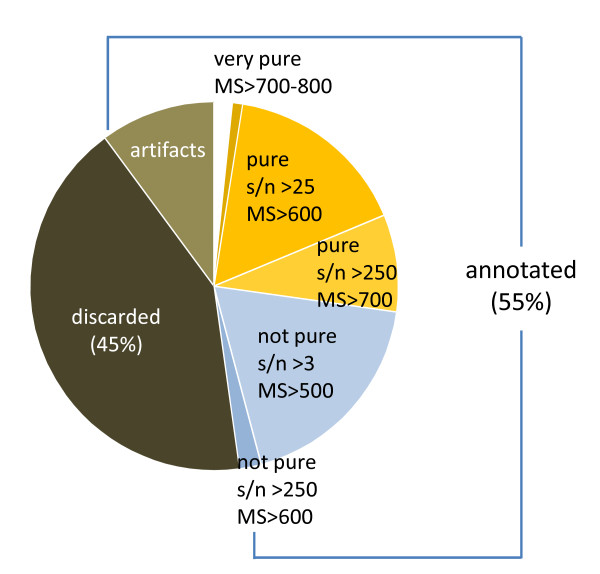
**Filtering effect of the vocBinBase algorithm**. Spectra must meet multiple criteria to be annotated and stored in the database. 45% of all incoming spectra fail to meet criteria and are discarded as noisy and inconsistent. The remaining 55% are annotated and stored in the database. 12% of annotated spectra are column or Twister™ polysiloxane artifacts. A further breakdown of annotated spectra based on peak purity, s/n, and mass spectral similarity is shown.

At present the database contains spectra from 3,435 samples representing 18 species. Despite the 1.7 million imported, fully deconvoluted spectra, the vocBinBase database currently only contains 1537 unique Bins. Of all imported spectra, 45% fail to meet algorithm thresholds and are discarded; such spectra are noisy and inconsistent. The lower users set thresholds for peak detections in ChromaTOF (e.g., lowering peak finding criteria from s/n>20 to s/n>3), the more peaks would be detected. Most of the corresponding peak spectra would be discarded by the BinBase algorithm as too noisy and not be reported in output sheets. A similar rate of discarding spectra was reported by the SpectConnect tool [25] that employs AMDIS deconvolution data [24] of GC-quadrupole MS instruments. Under the settings used here, the remaining 55% of the spectra meet the quality criteria and are annotated and stored in the database (Figure 5). Approximately 12% of the annotated compounds are column- and Twister™-derived polysiloxane artifacts; these artifacts are annotated by the algorithm but are not included in the BinBase reports exported for users. As described above, annotations rely on multiple criteria and certain thresholds are variable depending on various metadata values; the required MS similarity threshold depends on peak abundance and purity (e.g. a low purity peak requires a less stringent MS similarity match). A small percentage of annotated spectra (4%) are generated by very pure peaks (purity <0.15) with high MS similarity score, while the majority of database entries are generated by pure peaks (purity<1.5, 46%) or not pure peaks (purity>1.5, 39%).

**Figure 6:**
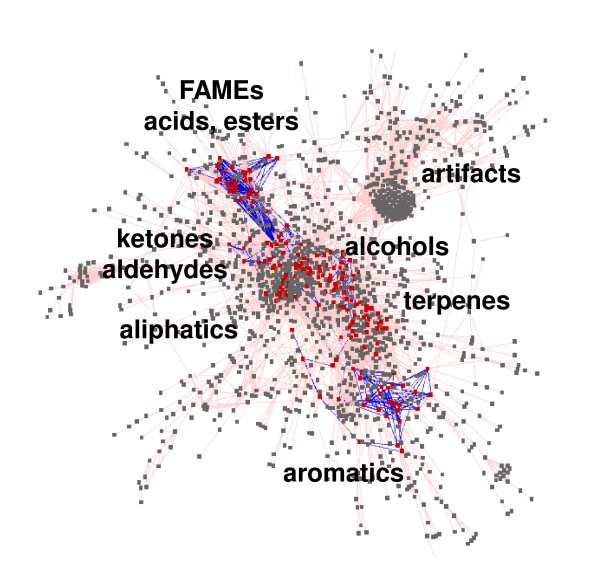
**Visualization of the vocBinBase database contents**. Red nodes are identified compounds, grey nodes are unidentified compounds. Blues edges link identified volatiles with structural similarity greater than 700.

Of the current 1,537 Bins, 211 have been identified as genuine volatiles through mass spectral-retention index matching. In addition, 161 Bins were annotated as polysiloxane artifacts (which therefore do not get exported into study result data sheets), and the remaining Bins are unidentified yet. Visualization of the VOC database contents using spectral similarity (all Bins) and the Tanimoto chemical similarity coefficient (identified Bins) was performed using Cytoscape (Figure 6). The Tanimoto similarity coefficient is a similarity metric that calculates a score indicating the level of similarity between molecules being compared [38]. The network overview provides a visual representation of the relationships between the 1537 Bins. The identified compounds are represented by red nodes and the unidentified compounds as grey nodes. Nodes clustered closely together are more similar than those nodes with just a single connection at the edge of the network. Blues edges link identified volatiles with structural similarity greater than 700. Note that the polysiloxane artifacts cluster away from the compounds, due to very distinctive fragmentation pattern. Network regions with identified compounds (red nodes) have been labeled with class information.

## Utility and Discussion

### User interfaces

#### SetupX/MiniX

SetupX is the Metabolomics Standards Initiative (MSI)-compliant study design database where researchers enter detailed information regarding experimental design in a standardized format prior to scheduling sample runs [35-37]. We have now developed a leaner version of this database, MiniX. MiniX utilizes biological parameters such as genotype, organ and treatment for delineating study classes. These experimental classes are subsequently used to create randomized data acquisition sequences, and are also linked to both new Bin generation and BinBase report table contents. Users schedule vocBinBase data annotation tasks through MiniX; multiple export options allow users to group experimental classes from multiple studies to facilitate cross-study analyses. BinBase annotation reports can be retrieved by all registered experimental collaborators through the MiniX web interface and can be made publically available once the data has been published. Other files related to an experiment can also be uploaded in MiniX for storage and sharing. In addition to serving as a repository for metadata and BinBase reports, MiniX provides an overview of scheduled experiments and current data acquisitions.

#### BinBase Compound Browser

The BinBase database stores and disseminates information regarding both identified and unidentified "Bins" or compounds. Apart from Bin queries through the vocBinBase download website (http://vocBinBase.fiehnlab.ucdavis.edu), both metabolite and volatile databases can be queried through the BinBase Compound Browser (http://eros.fiehnlab.ucdavis.edu:8080/binbase-compound/). Once the database has been selected, searches are conducted using name, database identifier or mass spectrum. Search results display key Bin properties including the Bin mass spectrum, unique database identifier, retention index, and quantitation ion. Identified compounds are displayed with the chemical name and PubChem identifier (hyperlinked to the appropriate webpage). All Bins are also displayed with a list of species in which the Bin has been detected, and a list of the ten most similar Bins in the database. This information can be useful in assigning putative compound classes or identifying likely shared substructures. The Compound Browser allows users to compare the queried Bin with any of the ten most similar Bins. See Additional File 3, figure S3, for sample screen shots detailing a Bin search.

#### Bellerophon

Bellerophon is the central administration tool for BinBase. Bin management (identification, quantification ion selections, export options, reference links) and database browsing are performed through Bellerophon. All registered users have access to full database content (can select, view and export data) but only specific users have rights to perform Bin maintenance.

A key function of Bellerophon is identifying Bins through library matching. This can be performed manually by selecting each Bin and initiating a search of the interfaced Adams library within the RI window and mass spectral match settings selected by the user. Additionally, an automated search option can be initiated, which highlights all Bins with library matches within specified RI and match score windows. A super user with Bin maintenance rights then manually checks the match and, if appropriate, adds the chemical name to the compound and links the Bin to external libraries such as KEGG or PubChem through the reference view. A sample search is provided in Additional File 4, figure S4. Several functions typically performed by MiniX have been duplicated in Bellerophon so that interruptions in MiniX service do not halt data acquisition or analysis workflows. Through Bellerophon, users can upload samples and assign sample classes, import data, initiate data annotation tasks and retrieve results files. Instructions for all of the above features are available at http://binbase.fiehnlab.ucdavis.edu:8080/confluence/display/BinBase/Bellerophon.

Contents of the vocBinBase database are publically available from http://vocBinBase.fiehnlab.ucdavis.edu (Figure 6) as downloads of mass spectra in the *.msp format for use in the NIST MS search program. The vocBinBase spectra library downloads contain both mass spectra and retention index data for all database Bins. Database entries are reported with both Fiehn FAME-based RI values and Kovats alkane-based RI values. In addition, annotation results of volatile profiles for a range of studies and species can be downloaded. Raw chromatogram data (either in Leco *.peg formats or as open access *.netcdf) are available on request.

#### Uses and benefits

Technological advances have facilitated comprehensive volatile compound monitoring in a wide range of research areas [16-23]. Whether discovering volatile biomarkers in human disease or researching multitrophic interactions in plant defense, the ability to find the signal in complex volatile mixtures remains a challenge. Specialized data processing tools are required to maximize the information extracted from the collected data.

The vocBinBase algorithm automates GC-TOF-MS chromatogram annotation in an efficient, reproducible, and robust manner that allows comparative analysis of diverse sample types over years. The algorithm assigns the identity of compounds existing in the database, and adds unique molecules to the database when a database match is not found. Because the database is continually updated as new compounds are detected, analysis is not dependent on a specific user library or commercially available compounds. By tracking unknowns we are not limited by user-injected standards or by compounds available for purchase, and can maintain a dynamically growing database. The database approach facilitates comprehensive data annotations for all studies. Although Bins are only created from high quality data, they can be matched at lower thresholds in samples where the compounds are not as abundant or are obscured by a neighboring peak or peaks.

vocBinBase is not specific to a sample source or sample collection method, and we anticipate this tool will be valuable in all areas of volatile research. The software is capable of annotating large datasets and is well-suited to cross-study comparisons (e.g., source, species, season). We have used vocBinBase in studies ranging from dozens to 1,727 samples per annotation query, from sources including plants and fruits, human breath, cigarette smoke and wine. Across the 3,435 samples analyzed to date 400 ± 200 (average ± standard deviation) peaks are reported by the Leco ChromaTOF of which 273 ± 110 fit the quality parameters required for annotation by the vocBinBase algorithm. Of the 400 annotated peaks in an experiment, around 80 to up to 170 unique volatiles were identified through vocBinBase mass spectral and retention index matching. Data analysis is fast, and depending on the sample complexity complete assignment can take seconds-to-minutes per chromatogram (e.g., 1,727 samples with 1,100 Bins required 2.2 min computation time per sample).

Well-annotated studies allow researchers to share, re-analyze and reuse data from multiple independent studies. The Fiehn vocBinBase database contents are publically available and mass spectra are reported with both FAME- and alkane-based RI information. Researchers have the ability to run mass spectral-RI searches of their data against our dynamic database. With the ability to probe hundreds of compounds across thousands of samples, researchers will be able to more efficiently use all data collected, and leverage knowledge in one field to aid discovery in another.

#### Comparison to similar databases

At present, there exist no similar peak-annotation/database tools available specifically for volatile compounds. The vocBinBase and the derivatized-metabolite BinBase annotation tools remain unique among other GC-TOF-MS annotation tools in incorporating a database approach. No other software stores information about unidentified compounds in addition to known compounds to be tracked and compared together in all subsequent experiments. This feature allows querying information about presence and abundance of novel (unknown) volatile metabolites across studies and species and thus enables researchers to prioritize identification efforts for structurally unassigned compounds.

A few publically accessible volatile compound databases have been constructed, but their utility is often limited by several factors. Most target a fairly specific research area, and limit the portion of volatile compound space covered by the individual database. The SuperScent database [33] provides structure and classification information for flavors and scents, Flavornet [32] features compounds identified in experiments employing gas chromatography-olfactometry (GC-O) analysis, and Pherobase [34] is focused on insect pheromones and semiochemicals. The examples listed here range in size from 738 compounds (Flavornet) to over 8,000 (Pherobase) and allow for searches by compound name, chemical class, or CAS number. Literature references, supplier information and retention index information are also captured. While Pherobase is growing, and others solicit information from the community at large, since 2004 Flavornet has not added additional volatile compounds to its database. Importantly, none of these databases provide mass spectral information in a manner that can be used conveniently in automated mass spectral library searches. With the Fiehn vocBinBase download (as .msp file), researchers are able to search against all Bins in a dynamically growing database.

#### Case study - essential oils

Replicate injections (n = 6) of eleven essential oils (Plantlife, San Clemente, CA) were performed to demonstrate vocBinBase annotation and databasing capabilities. Samples were diluted 100-fold in methylene chloride (Fisher Chemical, Fair Lawn, NJ) to prevent peak overloading and obtain the quality chromatograms required for new Bin generation. Full analysis of the 66 samples (including spectral validation, RI correction, annotation, new Bin generation and replacements) took 18 minutes, or 27 seconds per sample.

**Figure 7:**
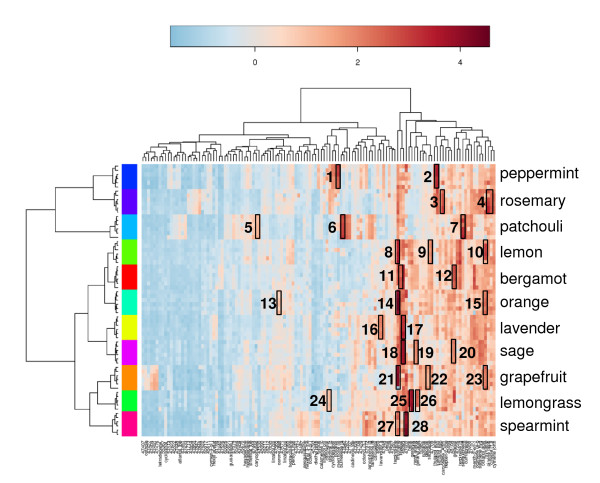
**Overview of volatile compound differences in 11 essential oil samples**. Key components of each oil are highlighted in the figure. Peppermint: menthone (1), menthol (2); Rosemary: camphene(3), α-pinene & 1,8-cineole (4); Patchouli: caryophyllene (5), bulnesene(6), terpinene (7); Lemon: limonene (8), sabinene (9), myrcene (10); Bergamot: linalool (11), geraniol (12); Orange: delta-3-carene (13), limonene (14), myrcene (15); Lavender: lavandulyl acetate (16), linalool (17); Sage: linalool (17), neryl acetate (18), geraniol (19); Grapefruit: limonene (20), sabinene (21), myrcene (22); Lemongrass: citronellal (23), neral (24), geranyl acetate (25); Spearmint: limonene (26), carvone (27).

The essential oil samples (rosemary, sage, bergamot, orange, lemon, grapefruit, patchouli, lavender, spearmint, peppermint) generated a total of 108 new Bins. Mass spectral-RI matching was performed on the newly-generated Bins through Bellerophon, which resulted in the annotation of 28 Bins to identified compounds. The vocBinBase-generated data report of 125 Bins (64 identified, 61 unidentified) was uploaded into the MetaboAnalyst web server for statistical analysis [39]. Hierarchical clustering was performed on the volatile data using the Pearson distance measure and Ward clustering algorithm. The resulting dendogram is shown along with a heatmap, which provides an overview of volatile compound differences among essential oil types (Figure 7). Key chemical constituents of each oil are highlighted in the figure [40].

## Conclusion

vocBinBase, a novel volatile compound annotation and database tool, has been developed, validated, and implemented. Standard methods have been devised for volatile sample collection, retention index marker addition, and GC-TOF-MS instrument operation. Integration of the Adams plant volatile library into the vocBinBase system was accomplished with a simple conversion of the Adams alkane-based RI data to its fatty acid methyl ester RI equivalents, which has accelerated compound identification through the Bin administration tool, Bellerophon. The vocBinBase database has annotated large datasets (hundreds to thousands of samples per study) and is well-suited to cross-study comparisons (e.g. source, species, season, etc.). To date, over 3,200 samples from 18 species have been analyzed in experiments ranging in size from 60-1,700 samples. The database currently contains mass spectral-retention index information for over 1,500 unique compounds, and is continuously growing. Database contents are available in Mass Search program format as a free resource for all volatile compound researchers (http://vocBinBase.fiehnlab.ucdavis.edu).

## Availability and requirements

The BinBase database code is available to the public at the Fiehn Lab website under the LGPL 2.0 license (http://binbase.sourceforge.net), and is accessible using different web front-ends and rich client applications as well as a webservice layer. Documentation required for installation and administration of the system is also found at this website. Database contents are available from http://vocBinBase.fiehnlab.ucdavis.edu.

## Authors' contributions

GW programmed all BinBase code, performed system validation and generated database statistics. KS developed sample collection methods, collected and analyzed all samples, and validated software performance in data analysis. DK performed calculations for the network Figure 6. OF conceptualized the project and supervised its development and completion. KS, GW and OF drafted the manuscript. All authors read and approved the final manuscript.

## Supplementary Material

Additional File 1 **The vocBinBase algorithm for annotation of GC-TOF-MS mass spectra (from **[35]). Figure S1. ChromaTOF metadata used in peak annotation include mass spectral similarity, peak purity (an estimate of the number, proximity and similarity of co-eluting peaks), retention index, signal-to-noise ratio, unique mass, and unique mass-to-base peak ratio. Click here for file

Additional File 2 **Conversion of retention index values between Adams and Fiehn chromatographic variants**. Figure S2. Alkane-based RI values supplied with the Adams library were converted to their Fiehn FAME-based RI value using a 2^nd ^order polynomial (A). The correlation between experimental and calculated FAME RI values for 70 injected standards (B) and for an additional 130 annotated Bins (C) is shown. A plot of the absolute RI deviation (RI_calculated_-RI_experimental_) is shown in (D). The standard deviation of the residual error is 3357 RI units (marked by dashed lines). Click here for file

Additional File 3 **BinBase compound browser web interface**. Figure S3. Database contents can be queried through the BinBase compound browser. A search for "linalool" (A) retrieves five database entries (B). Selection of 'linalool' (Bin 46027) directs the user to a page displaying Bin properties including mass spectrum, database identifier, RI and quantifier ion (C). Additional information regarding the ten most similar Bins and species data are also shown. Click here for file

Additional File 4 **Library search in Bellerophon**. Figure S4. In this screen shot, the Bellerophon Bin mass spectrum view (A), library mass spectrum view (B), Bin list view (C) and the similarity search view (D) have been configured for library matching and Bin annotation. Bin 46027 was generated from linalool standard injections. Double-clicking on the Bin populates the different views with the Bin mass spectrum and a list of library matches falling within the mass spectral match and RI windows set by the user (700 and 6000, respectively, in this example). Although seven library compounds fall within the match criteria, linalool is the highest quality match. Click here for file
