# Supplementary figures and images for: The volatile compound BinBase mass spectral database
Source: BMC Bioinformatics. 2011 Aug 4;12:321. doi: 10.1186/1471-2105-12-321 (PMC3199763; doi:10.1186/1471-2105-12-321)

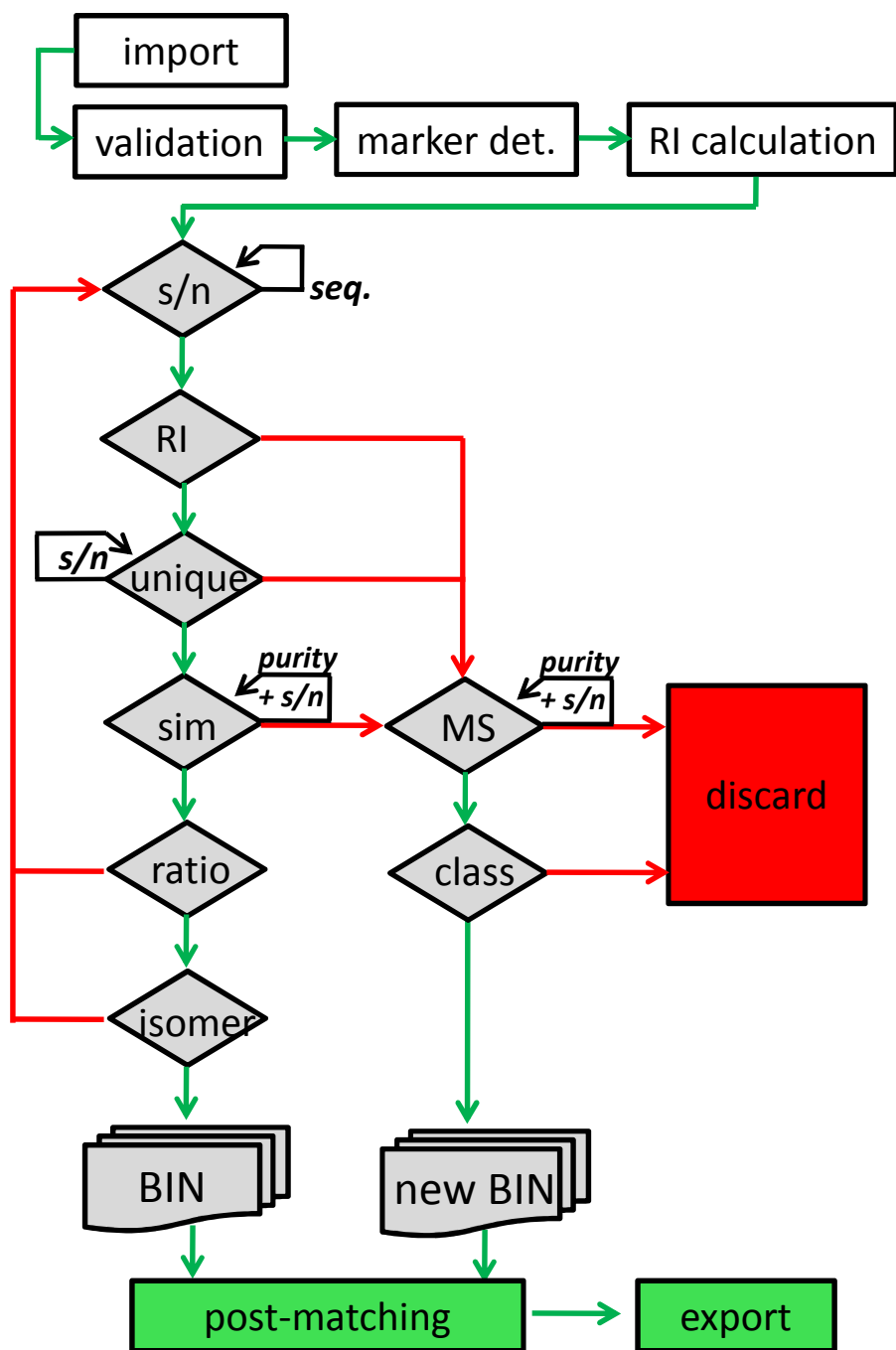

Supplement: Additional File 1 — The vocBinBase algorithm for annotation of GC-TOF-MS mass spectra (from [35]). Figure S1. ChromaTOF metadata used in peak annotation include mass spectral similarity, peak purity (an estimate of the number, proximity and similarity of co-eluting peaks), retention index, signal-to-noise ratio, unique mass, and unique mass-to-base peak ratio. [file 1471-2105-12-321-S1.PDF]

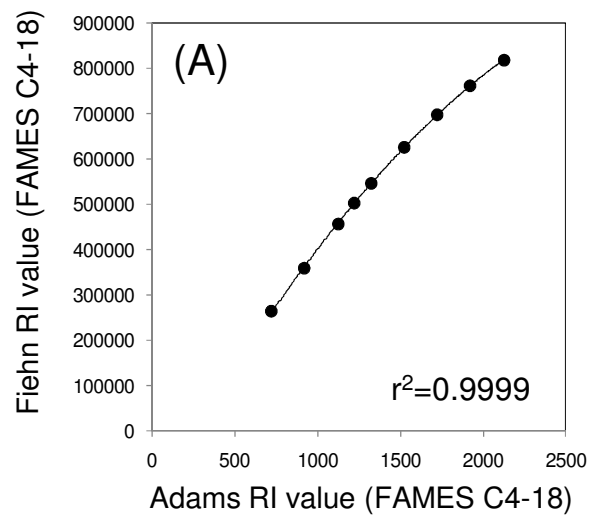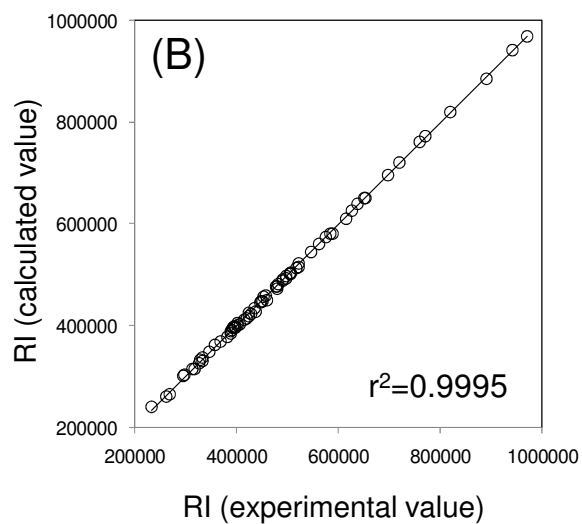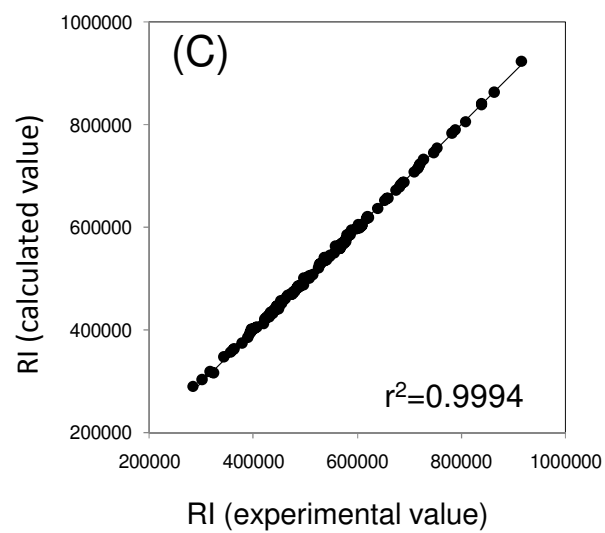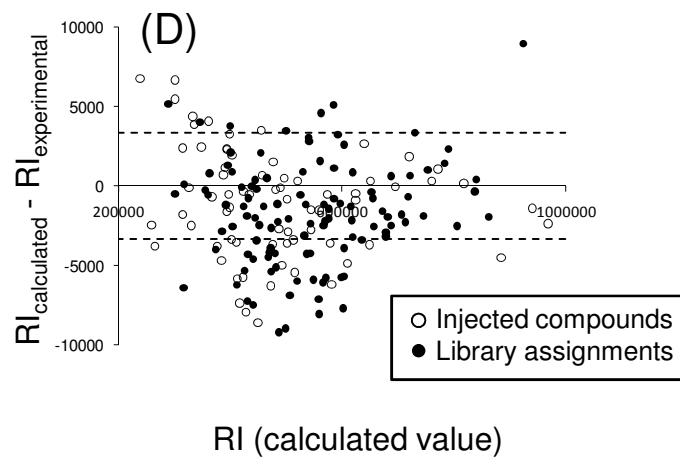

Supplement: Additional File 2 — Conversion of retention index values between Adams and Fiehn chromatographic variants. Figure S2. Alkane-based RI values supplied with the Adams library were converted to their Fiehn FAME-based RI value using a 2nd order polynomial (A). The correlation between experimental and calculated FAME RI values for 70 injected standards (B) and for an additional 130 annotated Bins (C) is shown. A plot of the absolute RI deviation (RIcalculated-RIexperimental) is shown in (D). The standard deviation of the residual error is 3357 RI units (marked by dashed lines). [file 1471-2105-12-321-S2.PDF]

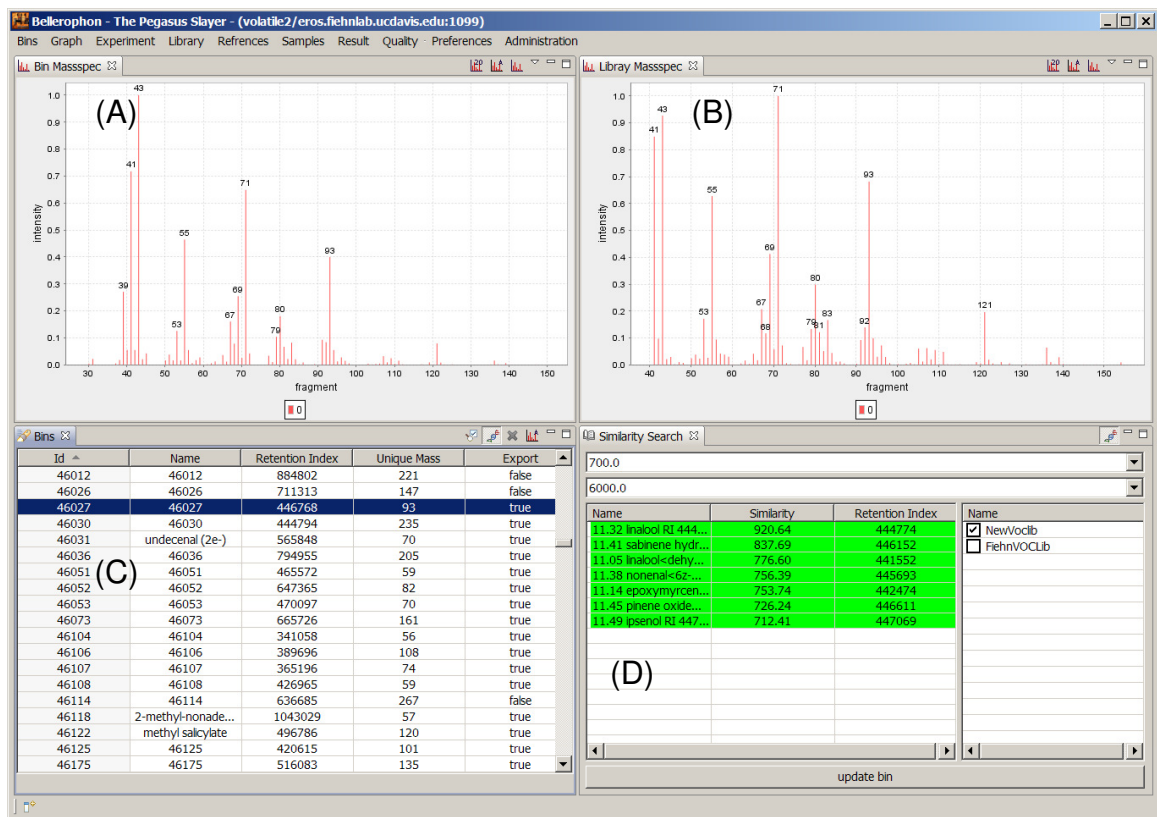

Supplement: Additional File 4 — Library search in Bellerophon. Figure S4. In this screen shot, the Bellerophon Bin mass spectrum view (A), library mass spectrum view (B), Bin list view (C) and the similarity search view (D) have been configured for library matching and Bin annotation. Bin 46027 was generated from linalool standard injections. Double-clicking on the Bin populates the different views with the Bin mass spectrum and a list of library matches falling within the mass spectral match and RI windows set by the user (700 and 6000, respectively, in this example). Although seven library compounds fall within the match criteria, linalool is the highest quality match. [file 1471-2105-12-321-S4.PDF]
