# Supplementary material for: The volatile compound BinBase mass spectral database
Source: BMC Bioinformatics. 2011 Aug 4;12:321. doi: 10.1186/1471-2105-12-321 (PMC3199763; doi:10.1186/1471-2105-12-321)
Supplement: Additional File 3 — BinBase compound browser web interface. Figure S3. Database contents can be queried through the BinBase compound browser. A search for "linalool" (A) retrieves five database entries (B). Selection of 'linalool' (Bin 46027) directs the user to a page displaying Bin properties including mass spectrum, database identifier, RI and quantifier ion (C). Additional information regarding the ten most similar Bins and species data are also shown. [file 1471-2105-12-321-S3.PDF]

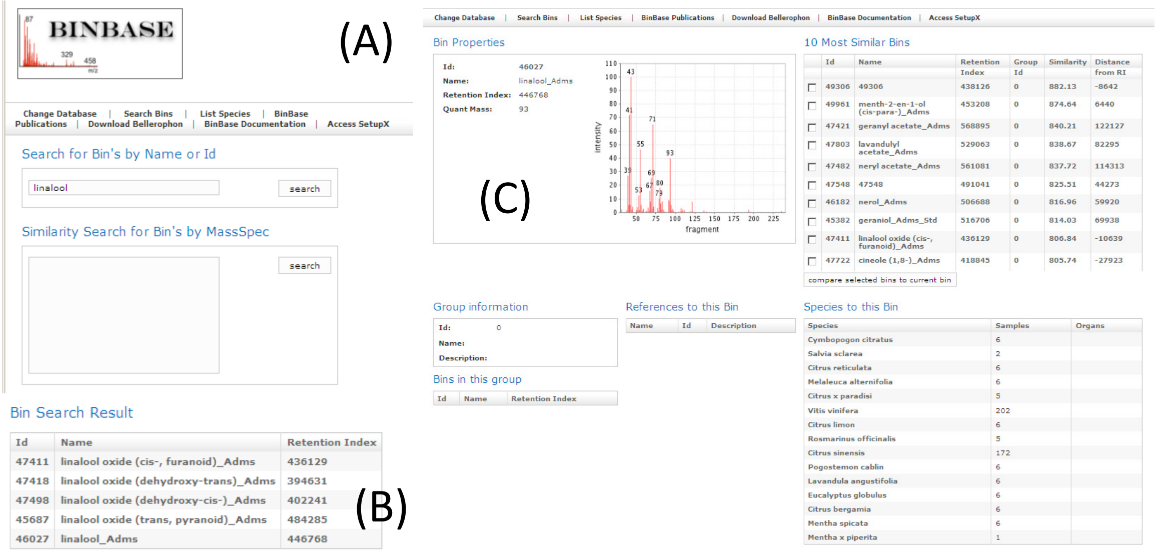

(A)
BINBASE
Change Database | Search Bins | List Species | BinBase Publications | Download Bellerophon | BinBase Documentation | Access SetupX
Search for Bin's by Name or Id
linalool
search
Similarity Search for Bin's by MassSpec
search
Bin Search Result
Id | Name | Retention Index
47411 | linalool oxide (cis-, furanoid)_Adms | 436129
47418 | linalool oxide (dehydroxy-trans)_Adms | 394631
47498 | linalool oxide (dehydroxy-cis-)_Adms | 402241
45687 | linalool oxide (trans, pyranoid)_Adms | 484285
46027 | linalool_Adms | 446768
(B)
Change Database | Search Bins | List Species | BinBase Publications | Download Bellerophon | BinBase Documentation | Access SetupX
Bin Properties
Id: 46027
Name: linalool_Adms
Retention Index: 446768
Quant Mass: 93
(C)
intensity
fragment
10 Most Similar Bins
Id | Name | Retention Index | Group Id | Similarity | Distance from RI
49306 | 49306 | 438126 | 0 | 882.13 | -8642
49961 | menth-2-en-1-ol (cis-para-)_Adms | 453208 | 0 | 874.64 | 6440
47421 | geranyl acetate_Adms | 568895 | 0 | 840.21 | 122127
47803 | lavandulyl acetate_Adms | 529063 | 0 | 838.67 | 82295
47482 | neryl acetate_Adms | 561081 | 0 | 837.72 | 114313
47548 | 47548 | 491041 | 0 | 825.51 | 44273
46182 | nerol_Adms | 506688 | 0 | 816.96 | 59920
45382 | geraniol_Adms_Std | 516706 | 0 | 814.03 | 69938
47411 | linalool oxide (cis-, furanoid)_Adms | 436129 | 0 | 806.84 | -10639
47722 | cineole (1,8-)_Adms | 418845 | 0 | 805.74 | -27923
compare selected bins to current bin
Group information
Id: 0
Name:
Description:
Bins in this group
Id | Name | Retention Index
References to this Bin
Name | Id | Description
Species to this Bin
Species | Samples | Organs
Cymbopogon citratus | 6
Salvia sclarea | 2
Citrus reticulata | 6
Melaleuca alternifolia | 6
Citrus x paradisi | 5
Vitis vinifera | 202
Citrus limon | 6
Rosmarinus officinalis | 5
Citrus sinensis | 172
Pogostemon cablin | 6
Lavandula angustifolia | 6
Eucalyptus globulus | 6
Citrus bergamia | 6
Mentha spicata | 6
Mentha x piperita | 1
